# Self-assembly of a parallelogram black phosphorus ribbon into a nanotube

**DOI:** 10.1038/s41598-017-13328-w

**Published:** 2017-10-11

**Authors:** Jiao Shi, Kun Cai, Ling-Nan Liu, Qing-Hua Qin

**Affiliations:** 1 College of Water Resources and Architectural Engineering, Northwest A&F University, Yangling, 712100 China; 2 Research School of Engineering, the Australian National University, ACT, Canberra, 2601 Australia

## Abstract

A nanotube from single-layer black phosphorus (BP) has never been discovered in experiments. The present study proposed a method for the fabrication of a BP nanotube (BPNT) from a parallelogram nanoribbon self-assembled on a carbon nanotube (CNT). The nanoribbon has a pair of opposite sides along the third principal direction. According to the numerical simulation via molecular dynamics approach, we discover that a wider BP nanoribbon can form into a series of chiral nanotube by self-assembly upon CNTs with different radii. The radius of a BPNT from the same ribbon has a wide range, and depends on both geometry of the ribbon and the CNT. One can obtain a BPNT with the specified radius by placing the ribbon nearby a given CNT. The method provides a clue for potential fabrication of BPNTs.

## Introduction

**Figure 1:**
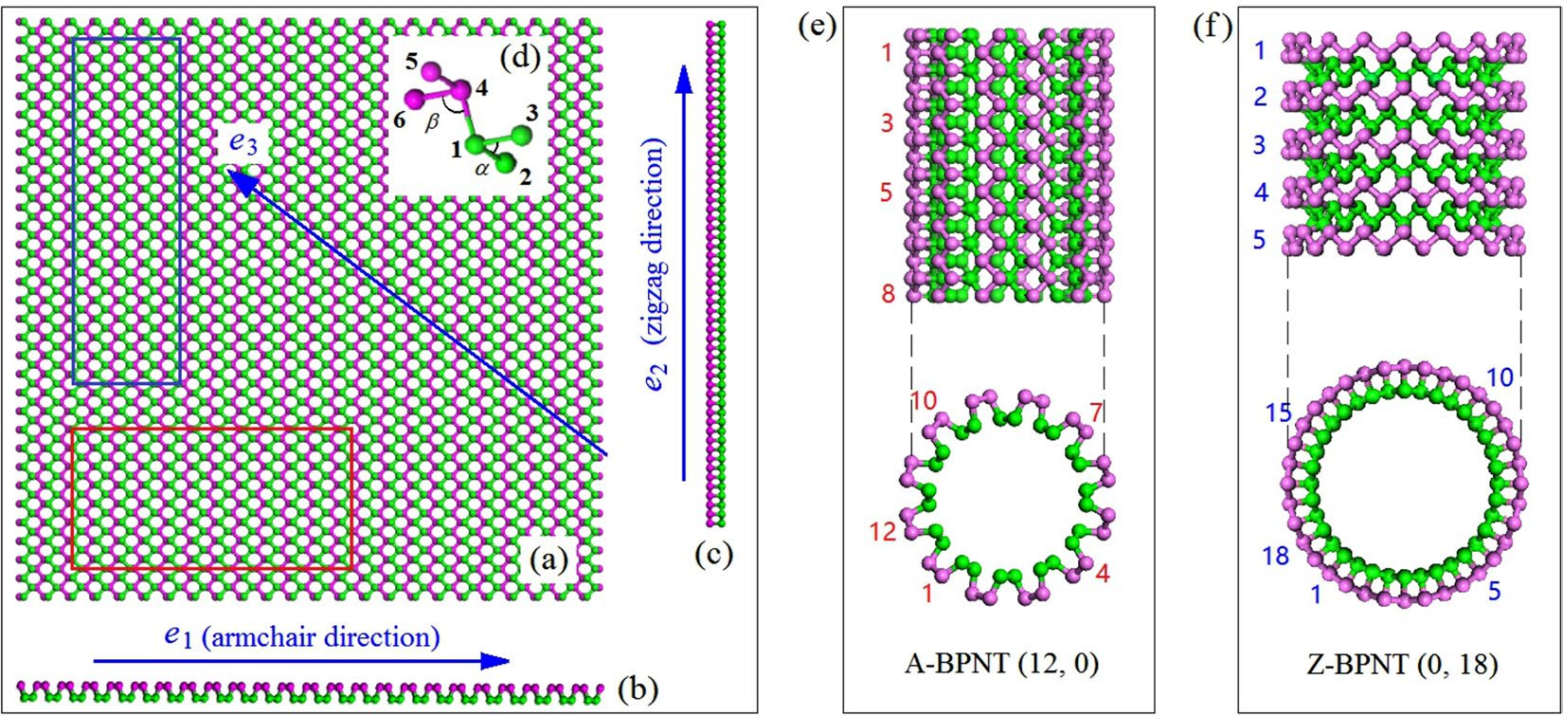
Geometry of a rectangular BP ribbon and possible nanotube from the ribbon with three principle directions, i.e., *e*_1_, *e*_2_ and *e*_3_. (**a**) Top view of SLBP; (**b**) zigzag-view of SLBP; (**c**) armchair-view of SLBP; (**d**) unit cell with bond lengths of *L*_12_ = *L*_13_ = *L*_45_ = *L*_46_ = 0.24244 nm, *L*_14_ = 0.23827 nm, and bond angles of *α* = 98.213°, *β* = 97.64°; (**e**) Armchair BPNT (A-BPNT), which is formed by curling the armchair sides of SLBP within the red rectangular in (a); (**f**) Zigzag BPNT(Z-BPNT), obtained by curling the zigzag sides of SLBP within the blue rectangle in (a). For simplicity, using (*N*_A_, *N*_Z_) labels the chirality of a BPNT, where *N*_A_ and *N*_Z_ are the numbers of periodic cells along the armchair and zigzag directions within the cross section, respectively, e.g., *N*_A_ = 12, *N*_Z_ = 0 in (e). For a chiral BPNT, both *N*_A_ and *N*_Z_ are different from zero.

Among many phosphorus (P) allotropes^1–3^, black phosphorus (BP)^4^ shows excellent electric properties at lower dimension, e.g., two-dimension (2D)^5–7^. Due to their excellent electric properties including direct band gap, high free carrier mobility at room temperature, and anisotropic electric conductance, few-layered BP becomes a competitive candidate of 2D materials in nanodevices. For a component in a nanodevice made from any material, its mechanical property is usually a basic index to show the stability of the nanosystem. In a few-layered BP, the neighbor P atoms within the same layer are covalently bonded via 3 sp^3^ hybrid orbitals, and the interaction between the P atoms in neighbor layers is van der Waals (vdW) interaction^8,9^. As the strength of P-P bond is moderately stronger than the vdW interaction in BP^10^, fabrication of a few-layered BP is much difficult than peeling a few-layered graphene from graphite^11,12^. Moreover, the atoms in a BP behave chemically active at each stage of the fabrication process and tend to be bonded with foreign atoms which are also chemically active . This is the major reason for the instability of BP exposed in air or water^13,14^. Relatively, the internal P atoms in BP appear more stable than the edge atoms. Hence, to protect a one-dimensional component made from BP, one can try to reduce the number of unsaturated edge atoms. Besides chemical approach by covalently bonded the atoms with other atoms^15,16^, the stability of a BP can be enhanced by changing the topology of the BP. For example, one can form a rectangular BP ribbon into a nanotube (Fig. 1e,f)^8,17–22^, whose electric property is excellent, too, according to the first-principle calculations^18^. As the opposite sides of a rectangular BP ribbon are covalently bonded together, those atoms on the two sides become as stable as the internal atoms.

Similarly, BP’s mechanical property is important to the design of nanodevices. Cai *et al*.^20^ estimated the stability of a free BP nanotube (BPNT) at finite temperature and found that the BPNT with lower radius collapse easier at the same temperature. It is also noted that a zigzag BPNT breaks easier than an armchair BPNT with the same radius at the same temperature. Following this line, Cai *et al*. also investigated the buckling behavior of a BPNT under uniaxial compression^21,23^. Shi *et al*.^24^ examined the strength of a BPNT in a rotating CNT. These studies found that the collapse of a nanotube can be induced by centrifugal force when the rotating speed of the CNT exceeds a critical value.

Unfortunately, a phosphorus nanotube has never been discovered in experiments up to now. Hence, people suggest to fabricating a nanotube from a single-layered BP using self-assembly approach^25–29^. For example, Cai *et al*.^30^ used a carbon nanotube^31,32^ to trigger the self-assembly of a rectangular BP ribbon. Their work reported a BPNT can only be obtained on some restricted conditions. For example, the BP ribbon should not be polluted via a fragment on the surface contacting CNT^33^. As the armchair edge of a BP ribbon looks like a wave line and can be curved much easier than that along its normal direction. Hence, in fabrication the length of the BP ribbon along armchair direction should be very close to 2*π*(*r* + 0.34)nm, where *r* is the radius of the CNT used to actuate the self-assembly of the ribbon. If the length is different from the value of 2π(*r* + 0.34)nm obviously, a nanotube cannot be formed from the BP ribbon. The reason is that the attraction between the BP nanostructure and the CNT is much strong, and the opposite sides parallel to the generator of CNT need to meet for covalent bonding. As we choose a parallelogram BP with a pair of opposite sides along the third principle direction (*e*_3_ in Fig. 1a), the oblique sides can be a helix as the BP wound upon the CNT. The helix angle may adapt to the radius of the CNT. To improve the success rate of self-assembly, in this study, we suggest a new scheme to fabricate a BPNT from a parallelogram ribbon, in which the opposite sides are parallel to the third principle direction, rather than the zigzag direction. Numerical experiments are carried out to verify the feasibility of the method. Results show that lots of chiral BPNTs with different radius can be formed from the same parallelogram ribbon.

## Models and Methodology

### Models

**Figure 2:**
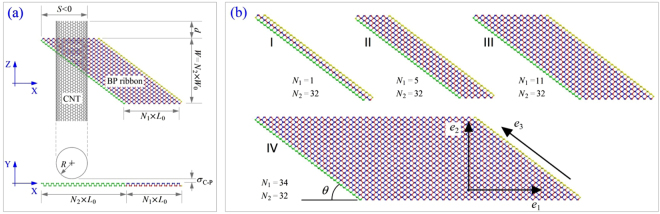
Geometry of the system including a carbon nanotube (CNT) and a parallelogram BP nanoribbon. (**a**) Relative position between BP ribbon and CNT. (b) Four BP samples involved in experiments. *S* is the X-direction distance between the right edge of CNT and the upper left tip of BP ribbon. Distance between upper edges of CNT and BP ribbon *d* = ~1.524 nm. The axial length of CNT is ~8.68 nm. Within 0.5 nm of each hydrogenated end of CNT, the atoms are fixed in simulation. *R* is the radius of CNT, *σ*_C-P_ (=~0.34 nm) is the surface distance between CNT and BP ribbon. *L*_0_ (=~0.44 nm) and *W*_0_ (=~0.33 nm) are the unit length of BP along armchair and zigzag direction, respectively. *θ* = 37.137°.

Two major reasons motivated us to choose CNT to actuate the self-assembly of the BP nanoribbon. One is that perfect CNTs with different radius are easily obtained and have been widely investigated for applications in various kinds of nanodevices^34–45^. Another is that the surface interaction between CNT and BP ribbon is weaker than that between two BP ribbons^10^. It means the BP nanotube/nanoscroll may be departed from the CNT. In Fig. 2a, *R*, the radius of CNT, will assume to be in a wide range in simulation. One of the four ribbons shown in Fig. 2b has the same value of *N*_2_ and will be placed nearby a CNT with a distance of *S* along X-direction. As the upper left corner of the BP ribbon is at the left side of the right edge of the CNT, *S* is negative, which means the ribbon requires shorter time to wind upon the CNT. As *S* is positive, the surface distance between the upper left tip of the ribbon and the CNT equals $\sqrt{{S}^{2}+{\sigma }_{{\rm{C}}-{\rm{P}}}^{2}}$, which means the value of *S* should be less than 0.94 nm, otherwise, the distance will be longer than the cutoff, i.e., 1.0 nm. If the surface distance is longer than the cutoff, there is no interaction between the CNT and the BP ribbon, and self-assembly of BP ribbon cannot be triggered. Meanwhile, if *S* is slight difference from 0.94 nm, the attraction of CNT on the ribbon will be too weak that needs longer time to complete the self-assembly process. Consider computational cost, we choose *S* ≤ 0.5nm in this study.

To avoid generating new *C*-*P* bonds between the CNT and the BP ribbon at the ends, the unsaturated carbon atoms at each end of the CNT is covalently bonded with hydrogen atoms. Besides the reduction of boundary effect at initial stage of self-assembly, we choose a CNT with axial length of about 2*d* + *W* (Fig. 2a). And we put the ribbon with *e*_2_ parallel to the generatrix of CNT because the bending stiffness along *e*_1_ direction is much lower than that along *e*_2_ or *e*_3_ direction^20^. Each P atom on the edge of ribbon is covalently bonded with two neighbor P atoms. Hence, although the edge atoms are unsaturated, they do not form dangling bonds. The configurations of the edges are relative stable before the generation of new P-P bonds.

### Methodology

#### Numerical experiment method

To illustrate the dynamic behavior of the system, we adopt molecular dynamics (MD) approach to update the positions of atoms step by step. The MD simulations are carried out by way with the open source code LAMMPS^46^. In the simulation, empirical potentials are used to describe the interactions among atoms. Briefly, the interaction between carbon and/or hydrogen atoms in the CNT involved in simulation is evaluated by the AIREBO potential^47^. The bonding action among neighbor phosphorus atoms is described using the Stillinger-Weber potential^48^ with the parameters recently given by Jiang^10^. The non-bonding interaction among atoms is estimated by Lennard-Jones (L-J) potential^49^, which can be expressed as

1 $${{\Pi }}_{ij}^{{\rm{LJ}}}=4{\varepsilon }_{ij}[{({\sigma }_{ij}/{r}_{ij})}^{12}-{({\sigma }_{ij}/{r}_{ij})}^{6}]$$

where *r*_*ij*_ is the spatial distance between atom *i* and atom *j*. Other parameters related to carbon, hydrogen, and phosphorus atoms are listed in Table 1.

**Table 1:** The L-J potential parameters between any pair of the carbon (C), hydrogen (H), and P atoms.

Atom i	Atom *j*	*σ*_*ij*_(nm)	*ε*_*ij*_(meV)
P	C	0.34225	6.878
P	P	0.3438	15.94
C	C	0.3400	2.844
C	H	0.3025	2.065
H	H	0.2650	1.499

Eight major steps are involved in a simulation, i.e.,

1. Build the parallelogram BP ribbons and a CNT with specified sizes;
2. Place CNTs nearby the BP ribbon with specified value of *d* & *S*;
3. Renew the positions of atoms in the system by minimization of potential energy using steepest decent method;
4. Fix both ends of the CNT (and lower right corner of BP ribbon if necessary);
5. Set the system under canonical (NVT) ensemble with T = 8 K and Nosé-Hoover thermostat is adopted^50,51^;
6. Run 200 ps for further relaxation if necessary;
7. Run at most 2000 ps and record data;
8. Stop for post-processing.

The time step for the integral is set at 0.001 ps.

#### The stability of the system

Besides by snapshot observation of the system, in a simulation, the state of the system can also be evaluated by using the value of variation of potential energy (VPE) of the system, which is obtained by subtracting the initial potential energy of the components from the potential energy of the current system, i.e.,

2 $$\text{VPE}(t)={P}_{{\rm{system}}}(t)-{P}_{{\rm{CNT}}}({t}_{0})-{P}_{{\rm{BP}}}({t}_{0})={P}_{{\rm{C}}-{\rm{P}}}^{{\rm{vdW}}}+{P}_{{\rm{P}}-{\rm{P}}}^{{\rm{new}}}+{P}_{{\rm{CNT}}}^{{\rm{Deform}}}+{P}_{{\rm{BP}}}^{{\rm{Deform}}}$$

where the first item at right side of the equation is the total potential energy of the system at time *t*, the second and the third items are the initial potential energy of the CNT and the BP nanostructure, respectively, as time starts from *t*_0_. The values of *P*_CNT_ and *P*_BP_(*t*_0_) are fixed when the components are chosen, the value of *P*_system_ drops continuously before approaching an equilibrium state, and the value of VPE drops simultaneously. The item of ${P}_{{\rm{C}}-{\rm{P}}}^{{\rm{vdW}}}$ is negative due to vdW interaction between the BP ribbon and the CNT, which is the difference between the total potential energy of the system and those of the two separated components at a given time. ${P}_{{\rm{P}}-{\rm{P}}}^{{\rm{new}}}$ is negative as new covalent P-P bonds are generated during winding. The item of ${P}_{{\rm{BP}}}^{{\rm{Deform}}}$ is always positive due to the deformation caused by the variation of lengths and angles of bonds from its natural values. In general, ${P}_{{\rm{CNT}}}^{{\rm{Deform}}}$ is negligible. In general, higher drop of the value of potential energy means more stable of the configuration of the system. Especially, when the opposite sides of the parallelogram BP ribbon meet together and new P-P bonds are generated between the two sides (e.g., the green and the yellow sides), the potential energy of the system has sharp decreasing (${P}_{P-P}^{{\rm{new}}}$ item), because each new P-P bond leads to ~0.66 eV of drop^30^.

It is known that the value of a system’s potential energy approaches the minimum when the system is at its most stable state. Hence, we confirm that the system is at a (local) stable state at the moment of the lowest value of VPE. In general, the value of VPE may fluctuate due to two reasons. One is because of the thermal vibration of the atoms in the system. During thermal vibration, the potential energy of each atom changes frequently, which influences the value of *P*_system_, the summation of the potential energy of all the atoms. Another is that the kinematic energy may still exist after getting rid of the thermal vibration item. The two reasons may mutually affected within the potential energy till all the energy dissipated completely.

## Results and Discussion

To reveal the size effect of a parallelogram BP ribbon on its stability of configuration after winding upon a CNT, totally 18 schemes are examined in numerical experiments, and the related parameters of the models are listed in Table 2. For all the BP ribbons, *N*_2_ is assumed to be 34 for the convenience of comparison among the numerical results. The results of the BP ribbon successfully forming a nanotube can be easily found in the table. For example, when CNTs (8, 8), (7, 7) and (6, 6) are used for the winding of the BP ribbon with *N*_1_ = 11, the ribbon can form into a nanotube with different chirality. Similarly, the BP ribbon with *N*_1_ = 34 can also form into a nanotube on a CNT with large radius, e.g., CNT (32, 32), (35, 35) or (37, 37). Detailed discussions are given below with respect to the value of *N*_1_ of a parallelogram ribbon.

**Table 2:** Parameters for different models with number of P atoms in ribbon and a CNT with different length. Marks ‘Yes’ or ‘No’ in ‘Success’ indicate if a ribbon successfully or unsuccessfully forms into a nanotube, respectively. “*” means 200 ps of relaxation before releasing BP ribbon.

BP ribbon	P atoms	CNT	*R*/nm	*S*/nm	Success?	BP ribbon	P atoms	CNT	*R*/nm	*S*/nm	Success?
I: *N*_1_ = 1	204	(5, 5)	0.339	0	No	II: *N*_1_ = 5	484	(5, 5)	0.339	0	No
		(20, 20)	1.356	0	No			(20, 20)	1.356	0	No
III: *N*_1_ = 11	904	(20, 20)	1.356	−3.98	No*	IV: *N*_1_ = 34	2514	(20, 20)	1.356	0.5	No
		(20, 20)	1.356	−3.98	No			(25, 25)	1.695	0.5	No
		(10, 10)	0.678	0.5	No			(28, 28)	1.799	0.5	No
		(10, 10)	0.678	0	No			(30, 30)	2.034	0.5	No
		(8, 8)	0.529	0	**Yes**			(32, 32)	2.170	0.5	**Yes**
		(7, 7)	0.475	0	**Yes**			(35, 35)	2.373	0.5	**Yes**
		(6, 6)	0.407	0	**Yes**			(37, 37)	2.509	0.5	**Yes**

### Winding of BP ribbon with *N*_1_ = 1 on CNTs

**Figure 3:**
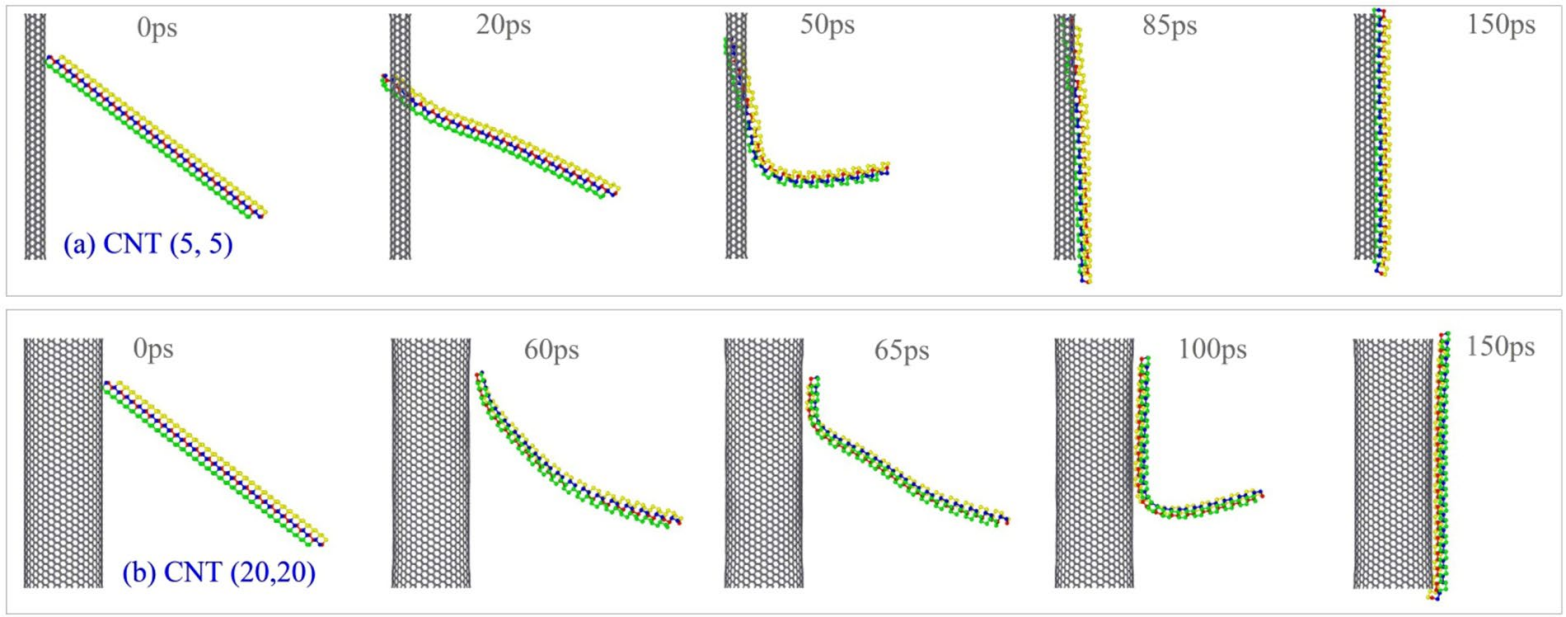
Sequential snapshots of a BP ribbon with *N*_1_ = 1 and *S* = 0 during winding on CNTs. (**a**) On CNT (5, 5), (**b**) on CNT (20, 20).

**Figure 4:**
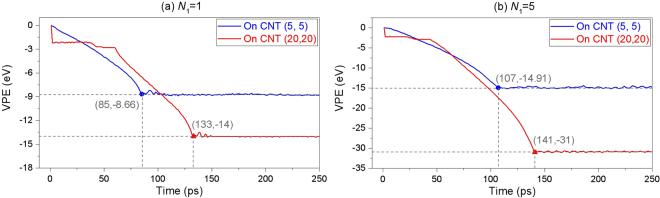
Histories of VPE of the system when a BP ribbon is winding upon a CNT. (**a**) The BP ribbon with *N*_1_ = 1, (**b**) *N*_1_ = 5. The solid symbols are marked to show the corresponding time and value of VPE.

A question is, if, for example, the width of a parallelogram BP ribbon is very small, e.g., *N*_1_ = 1, can it be attracted by and winding upon a CNT? Here, we test it by means of two CNTs, i.e., (5, 5) with radius of 0.339 nm and (20, 20) with radius of 1.356 nm. According to the molecular dynamics simulation results, the sequential snapshots of the BP ribbon on both CNTs are given in Fig. 3. One can find that the thin ribbon is attracted upon the outer surface of CNT after about 150 ps (Movie 1). The ribbon does not wind into a circular on the outer surface of CNT. Two factors lead to the phenomenon. One is that the attraction between C and P atoms is very strong, at 8 K, the thermal vibration of atoms cannot repulse the ribbon away from the CNT. Another is that the bending of such thin BP ribbon along *e*_2_ or *e*_3_ will result in increasing of the potential energy of the system, which violates the rule of minimum of potential energy of the system at a stable state, i.e., in Eq. (2), ${P}_{{\rm{C}}-{\rm{P}}}^{{\rm{vdW}}}$ + ${P}_{{\rm{BP}}}^{{\rm{Deform}}}$ > 0. Hence, the ribbon can only attach to the outer surface of the CNT along a generatrix. It also implies that one cannot form curved BP nanoscroll by self-assembly a slim BP ribbon upon a CNT. The VPE of the system with CNT (5, 5) (Fig. 4a) is lower than that of the system with CNT (20, 20) due to higher curvature the ribbon on CNT (5, 5).

### Winding of BP ribbon with *N*_1_ = 5 on CNTs

**Figure 5:**
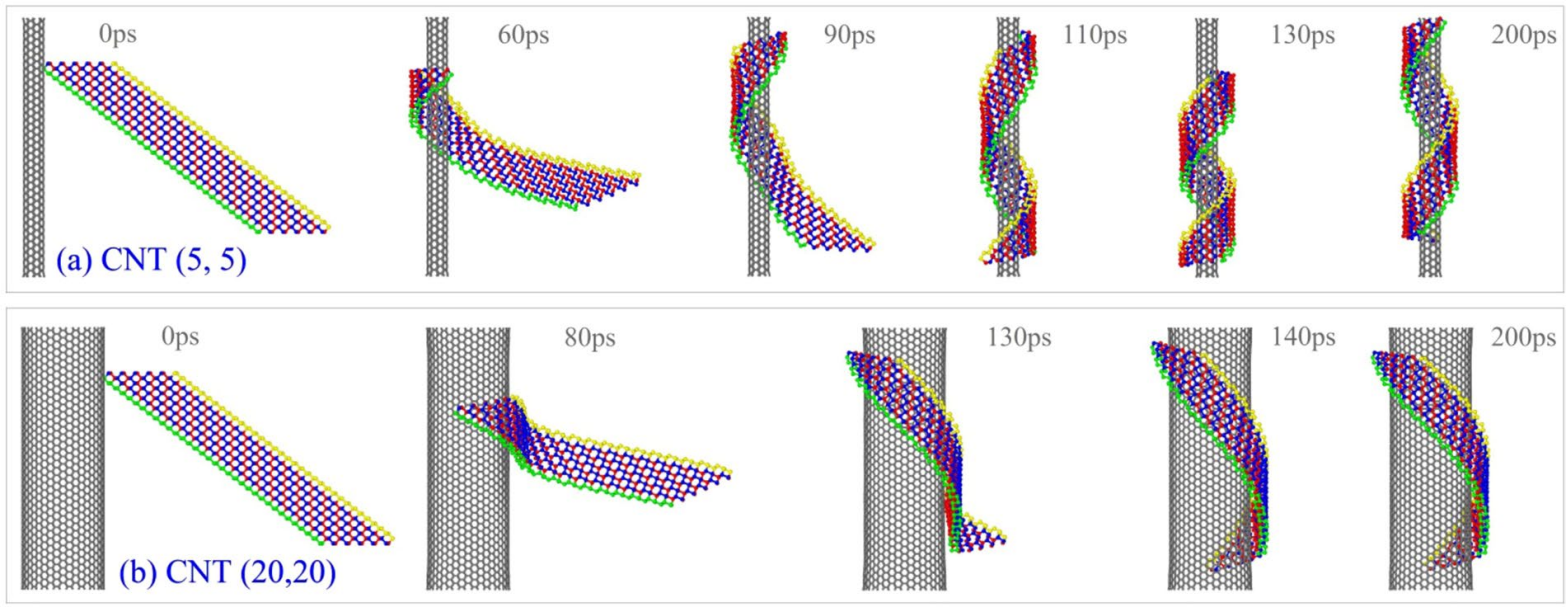
Sequential snapshots of a BP ribbon with *N*_1_ = 5 and *S* = 0 during winding on CNTs. (**a**) On CNT (5, 5), (**b**) on CNT (20, 20).

If we choose a BP ribbon with *N*_1_ = 5, which means the ribbon is much wider than the ribbon with *N*_1_ = 1, and put it nearby both CNTs, i.e., (5, 5) and (20, 20), the ribbon can form into neither a nanoscroll nor a nanotube. The representative snapshots of the system are shown in Fig. 5. Obviously, the ribbon becomes curved and attaches to the upper surface of CNT. It is because the vdW interaction between the ribbon and CNT is far greater than that of bending stiffness of the ribbon along *e*_1_ (armchair) direction, i.e., in Eq. (2), ${P}_{{\rm{C}}-{\rm{P}}}^{{\rm{vdW}}}$ + ${P}_{{\rm{BP}}}^{{\rm{Deform}}}\ll 0$.

Having compared the two groups of snapshots in Fig. 5, one can find the difference of their final stable configurations of the BP ribbon on both CNTs. Due to the same helix angle (*θ*) of BP ribbon at initial stage, the ribbon has about 1.5 rounds of winding upon the surface of CNT (5, 5), whilst, less than 1 round upon CNT (20, 20). This phenomenon inspires a prediction, i.e., one can obtain a nanotube from the BP ribbon with larger value of *N*_1_ upon a CNT with a range of radius, or one can obtain a BP nanoscroll by winding a wider BP ribbon on a slim CNT. On the other hand, the slight difference between the snapshots at 140 ps and 200 ps of the ribbon on CNT (20, 20) (Fig. 5b) implies that the attraction of the surfaces between the ribbon and the CNT is much strong, and the ribbon moves hardly on the surface. However, one can find that the differences among the snapshots at 110 ps, 130 ps and 200 ps of the ribbon on CNT (5, 5) (Fig. 5a) implies that the surface attraction between the ribbon and the CNT is weaker than that on CNT (20, 20) because the curve ribbon can slide and rotate on the thin CNT (Movie 2). The reason is that the curvature of the ribbon on the thin CNT is higher, which implies that the ribbon has higher potential energy with respect to deformation. This can be verified from Fig. 4b, in which the VPE of the ribbon on CNT (5, 5) is only ~−14.9 eV, whilst, ~−31eV when upon CNT (20, 20). As the ribbon is curved, the P-P bonds in the inner surface (closer to CNT) are under compression and the P-P bonds in the outer surface is under tension^20,21^. Controlled by the minimal potential energy of the system, the ribbon cannot have a larger curvature which makes the curved BP ribbon attaching to the thin CNT tightly. Hence, by using a slimmer CNT, one may obtain a BPNT from ribbon and can depart the BP ribbon from CNT more easily due to weaker interaction.

### Winding of BP ribbon with *N*_1_ = 11 on CNTs

**Figure 6:**
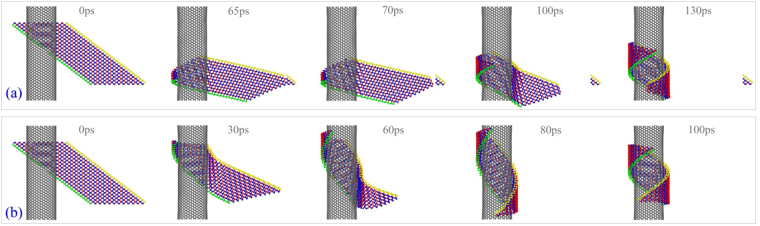
Sequential snapshots of a BP ribbon with *N*_1_ = 11 and *S* = −3.98 nm during winding on CNT (20, 20). (**a**) BP has 200 ps of relaxation before being released, (**b**) BP has no relaxation.

First, we put the BP ribbon with *N*_1_ = 11 and *S* = −3.98 nm nearby CNT (20,20) and observe the winding process. We find that the ribbon is torn at its lower right corner which is fixed during relaxation. The reason is that the strong attraction of CNT on the ribbon stretches the ribbon and the tension of the P-P bonds at upper side of the corner is too high (Movie 3). As the tension exceeds the strength of P-P bond, the bond is broken, and the breakage expands rapidly, e.g., starts from 65 ps and ends at 70 ps. Even before the end of 200 ps of relaxation, the major part of the BP ribbon has attracted to and wound upon the outer surface of CNT (20, 20) as shown in Fig. 6a. Hence, we do not provide more relaxation before releasing the ribbon in the other simulations. One can find that the BP ribbon has a similar stable configuration after being attached upon CNT (Fig. 6b).

**Figure 7:**
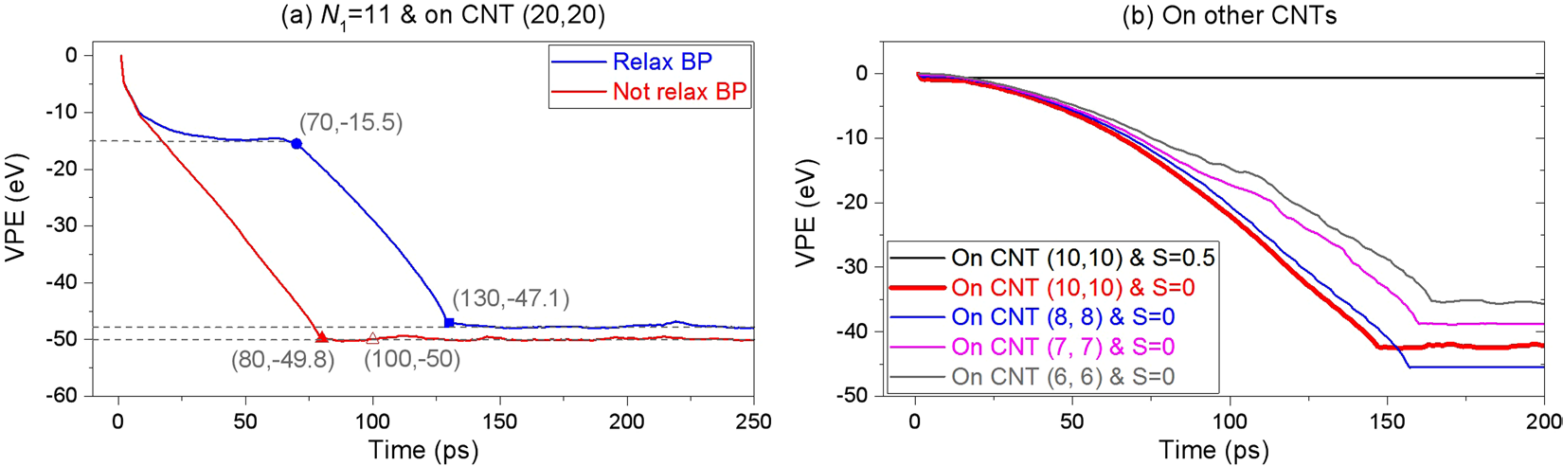
Histories of VPE of the system having the BP ribbon with *N*_1_ = 11 during winding upon a CNT. (**a**) On CNT (20, 20) with or without relaxation of ribbon when *S* = −3.98 nm. (**b**) On CNTs with lower radii. The solid symbols are marked to show the corresponding time and value of VPE.

As comparing the variation histories of the potential energy of the system with or without relaxation, the obvious difference between the two curves shown in Fig. 7a can be found. For example, the curve with respect to relaxation case has two stage of drop, i.e., during 0 and 70 ps, the potential energy of the system drops ~15.5 eV. Which is caused by both the fracture of the P-P bonds at the lower right corner and the increasing of the attach area between the ribbon and the CNT, and during 71 and 130 ps, the drop of potential energy is mainly caused by the interaction between the ribbon and CNT. The deformation of the ribbon, which leads to increasing of potential energy, has been considered simultaneously. As there is no relaxation, the BP ribbon is wholly moving towards the CNT at start. And the ribbon needs only about 80 ps to approach the stable state and the total decreasing of the potential energy of the system is ~50 eV, which is different from that of the system with relaxation. The difference of ~2.9 eV, is majorly because the lower right corner of the ribbon is not broken and is attracted to the outer surface of the CNT, simultaneously.

From the data shown in Fig. 7a, we know that the potential energy drops greater at 100 ps, which means that the configuration is more stable than that at 80 ps. As comparing the two snapshots at 80 and 100 ps in Fig. 6b, we find that the green edge and the yellow edge of the ribbon become closer at 100 ps. It hints that the two edges can meet on condition that either increasing the value of *N*_1_ or decreasing the radius of CNT. Based on this idea, we put the ribbon nearby other CNTs with lower radii, e.g., CNT (10, 10), (8, 8), (7, 7) or even (6, 6). Simulation results (Table 2) show that the ribbon can form into a nanotube on the three slimmer CNTs. And the VPE histories shown in Fig. 7b illustrate that the system has the lowest potential energy as the ribbon is wound upon CNT (8, 8). Meanwhile, the potential energy of the system with CNT (7, 7) drops larger than that with CNT (6, 6). The reason is that the deformation potential energy of the ribbon wound upon a CNT with lower radius increases. From Fig. 7b, one can also find that the VPE is a small negative constant when the ribbon is placed 0.5 nm away from CNT (10, 10). The constant is caused by the relaxation of both components in the system, and the ribbon is not attracted to and wound upon the CNT.

**Figure 8:**
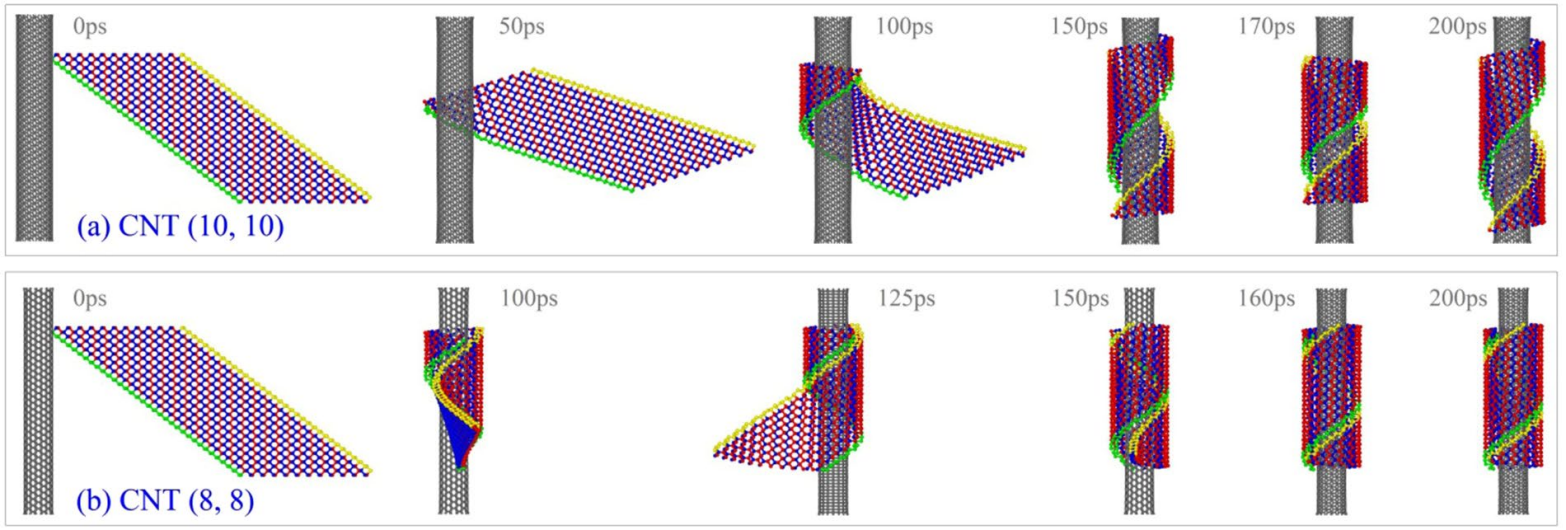
Sequential snapshots of a BP ribbon with *N*_1_ = 11 and *S* = 0 during winding on CNTs. (**a**) On CNT (10, 10), (**b**) on CNT (8, 8).

In Fig. 7b, the final value of the VPE of the system with respect to CNT (10, 10) and *S* = 0 is ~2 eV higher than that with respect to CNT (8, 8) and *S* = 0. The difference is caused by two factors. One is that the curvature of CNT (8, 8) is larger than that of CNT (10, 10) (see Fig. 8), which means the ribbon needs less potential energy to support its smaller deformation. Another is that the ribbon becomes a nanotube on CNT (8, 8), and the new P-P bonds between the yellow and the green edges lead to larger decreasing of potential energy (Movie 4). Clearly, the decreasing of potential energy caused by the generation of new P-P bonds is more than 2 eV due to stronger deformation of ribbon.

As the radii difference between CNTs (6, 6) and (8, 8) is ~0.122 nm, it means that the chirality of the BPNT is different on different CNTs. We can also learn from this phenomenon that a BPNT with specified radius can be formed by winding the same ribbon upon a given CNT. To verify this prediction, we choose a BP ribbon with larger value of *N*_1_ in next group of simulation.

### Winding of BP ribbon with *N*_1_ = 34 on CNTs

**Figure 9:**
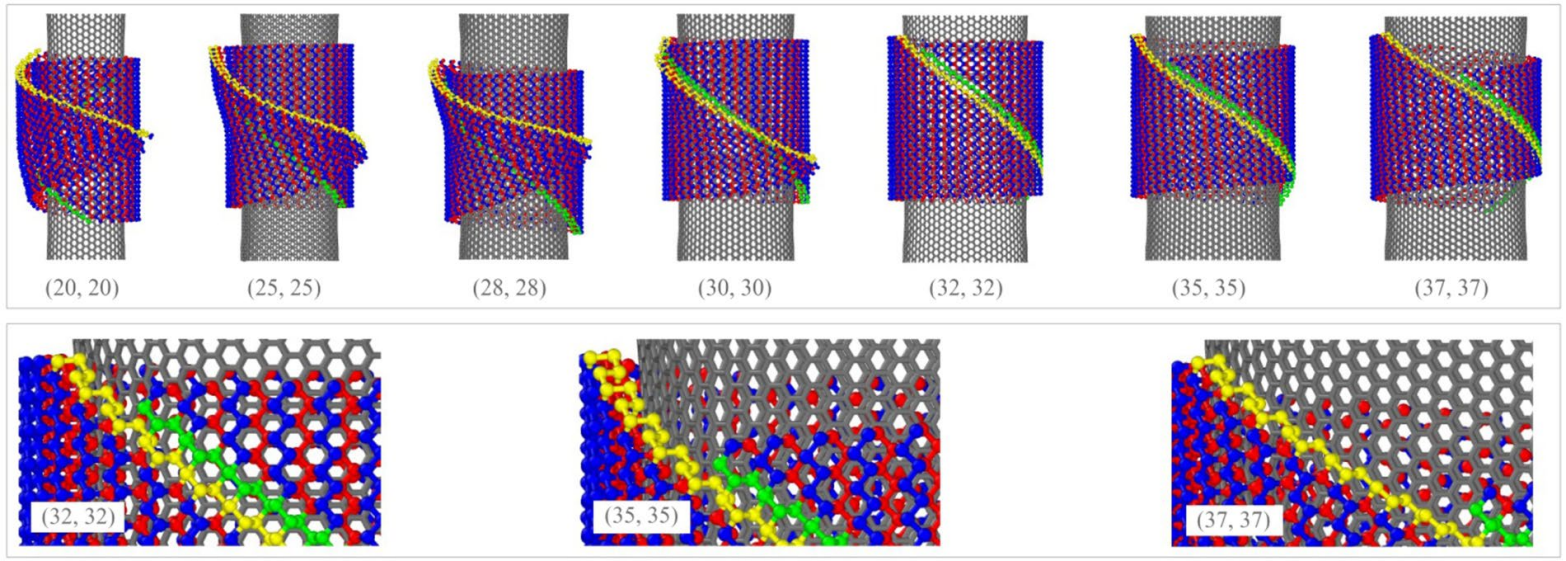
Final stable configuration of the same BP with *N*_1_ = 34 and *S* = 0.5 on CNTs. The lower three inserts are enlarged local configurations of BPNTs on different CNTs with higher radius.

To show the diversity of the winding results of a wider parallelogram BP ribbon upon CNTs with different radii, here we put the BP ribbon with *N*_1_ = 34 nearby one of the following CNTs, i.e., (20, 20), (25, 25), (28, 28), (30, 30), (32, 32), (35, 35) and (37, 37). And the final stable configurations of the BP structure are shown in Fig. 9.

From Fig. 9, we know that the wider ribbon becomes a nanoscroll on a CNT with lower radius, e.g., (30, 30). And the self-overlap of the BP nanoscroll is larger when the radius of CNT is smaller. But we also find that part of the green and the yellow edges of the ribbon are covalently bonded when they are wound upon CNT (30, 30). Hence, the BP ribbon with the minimum radius can be formed on a CNT with radius slightly larger than that of (30, 30). And the chirality of the BP ribbon is (*N*_1_ + 1, 0), i.e., an armchair nanotube.

**Figure 10:**
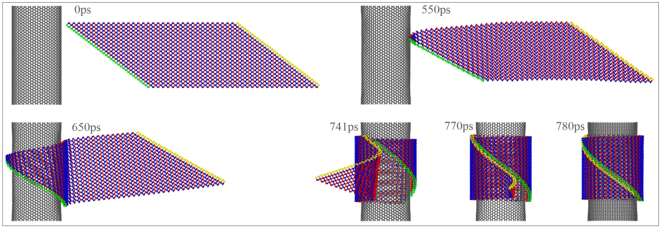
Sequential snapshots of a BP ribbon with *N*_1_ = 34 and *S* = 0.5 during winding on CNT (32, 32).

When putting the ribbon nearby a CNT with larger radius, e.g., (32, 32), the ribbon is finally formed into a nanotube and the representative snapshots are shown in Fig. 10 (Movie 5). According to both Table 2 and Fig. 9, the ribbon can be formed into a nanotube on CNTs of (35, 35) and (37, 37), as well. But by amplifying the upper parts of the BPNTs (the lower layer of Fig. 9), we find that their chirality are obviously different. For example, the upper ends of the green and the yellow edges have a dislocation of two periodic units along *e*_3_ direction (Fig. 2) on CNT (32, 32). According to the definition of BPNT’s chirality shown in Fig. 1, the chirality of the BPNT should be (*N*_1_ + 1 + 2, 2) = (37, 2), i.e., the numbers of unit cells along *e*_1_ and *e*_2_ directions are 37 and 2, respectively. Similarly, the chirality of the BPNT on CNT (35, 35) is (39, 4), or (41, 6) when upon CNT (37, 37). We find that the dislocation of the green and the yellow edges becomes higher when wound upon a CNT with larger radius. Obviously, the radii differences among the three BPNTs are obvious. Actually, we can also predict that a BPNT with larger radius can also be formed when putting it nearby a CNT with larger radius. When the dislocation is close to *N*_2_, the radius of the BPNT reaches its maximum. Hence, the radius of the BPNT from the same ribbon has a wider range than that by a rectangular BP ribbon^30^, i.e., we don’t need to prepare a BP ribbon with perfect length.

## Conclusions

By molecular dynamics simulation approach, we study the self-assembly of a parallelogram BP ribbon with opposite edges along the third principal direction on a CNT. According to the numerical results with respect to the different width of BP ribbons and different radii of CNTs, we find the ribbon can be formed into a nanotube in certain conditions. Some conclusions are drawn from the results for potential fabrication of a BPNT from a parallelogram ribbon.

- For a slim BP ribbon, it will attach to the outer surface of a CNT along the generatrix direction, and can form neither a BP nanoscroll nor a nanotube by self-assembly upon the CNT;
- If a BP ribbon with smaller value of *N*_1_ can be wound and further formed into a nanotube upon a CNT by covalently bonding between the P atoms on the opposite sides of the parallelogram along the third principal direction, the interaction between the BPNT and the CNT with shorter radius is smaller due to higher curvature of tube, and the BPNT can depart from the CNT more easily due to weaker interaction;
- For a BP ribbon with the same high value of *N*_1_, it can be formed into a series of chiral nanotubes on different CNTs with longer radii. The BPNT with the shortest radius should be an armchair type with chirality of (*N*_1_ + 1, 0). The BPNT with the longest radius depends both on the radius of CNT and on *N*_1_ + *N*_2_, and its chirality could approach (*N*_1_ + *N*_2_, *N*_2_ − 1), i.e., only one unit on each edge along the third direction are bonded together.

Some factors which are significant for experiment of self-assembly of a BP nanotube from a parallelogram nanoribbon are mentioned here. First, the corners of a parallelogram BP ribbon are not stable. From Fig. 9, we know that both ends of the chiral nanotube are smooth. As we need a nanotube with smooth ends, we can cut them by electron beams after self-assembly. Second, temperature involved in the present study is 8 K. Self-assembly at temperature higher than 100 K will be reported in our future work. Final, the system is in vacuum environment, and there are no other atoms except C and P in the present study. The unsaturated edges are not stable, especially at high temperature. Protecting air may be introduced to improve the stability. As these factors have been verified to be controllable, the feasibility of the present method is confirmed.

## Electronic supplementary material

Movie information

Movie 1--N1=1 on CNT (5,5) during[0, 150]ps.avi

Movie 2--N1=5 on CNT (5,5) during[0, 200]ps.avi

Movie 3--N1=11 on CNT (20,20) during[0, 200]ps after relaxation.avi

Movie 4--N1=11 on CNT (8,8) during[0, 200]ps.avi

Movie 5--N1=34 on CNT (32,32) during[580, 780]ps.avi
