# Supplementary material for: Self-assembly of a parallelogram black phosphorus ribbon into a nanotube
Source: Sci Rep. 2017 Oct 11;7:12951. doi: 10.1038/s41598-017-13328-w (PMC5636844; doi:10.1038/s41598-017-13328-w)
Supplement: Supplementary file 1 — Movie information [file 41598_2017_13328_MOESM1_ESM.pdf]

Self-assembly of a parallelogram black phosphorus ribbon into a nanotube

Jiao Shi [1], Kun Cai [1, 2*], Ling-Nan Liu [1], Qing-Hua Qin [2*]

[1] *College of Water Resources and Architectural Engineering, Northwest A&F University, Yangling 712100, China*

[2] *Research School of Engineering, the Australian National University, ACT, 2601, Australia*

* Author to whom correspondence should be addressed. Email: kuncai99@163.com (K. Cai); qinghua.qin@anu.edu.au (Q.H. Qin)

Supporting materials

Movies:

Movie 1--N1=1 on CNT (5,5) during[0, 150]ps.avi

Movie 2--N1=5 on CNT (5,5) during[0, 200]ps.avi

Movie 3--N1=11 on CNT (20,20) during[0, 200]ps after relaxation.avi

Movie 4--N1=11 on CNT (8,8) during[0, 200]ps.avi

Movie 5--N1=34 on CNT (32,32) during[580, 780]ps.avi
